# Extended Cystogastrostomy with Hydrogen Peroxide Irrigation Facilitates Endoscopic Pancreatic Necrosectomy

**DOI:** 10.1155/2017/7145803

**Published:** 2017-09-05

**Authors:** Mohamed O. Othman, Sherif Elhanafi, Mohammed Saadi, Christine Yu, Brian R. Davis

**Affiliations:** ^1^Gastroenterology and Hepatology Division, Baylor College of Medicine, Houston, TX, USA; ^2^Department of Medicine, Texas Tech University Health Sciences Center, Paul L. Foster School of Medicine, El Paso, TX, USA; ^3^Department of Surgery, Texas Tech University Health Sciences Center, Paul L. Foster School of Medicine, El Paso, TX, USA

## Abstract

**Introduction:**

Walled-off pancreatic necrosis (WOPN) is a major complication of acute pancreatitis. We hypothesized that an extended (2 cm) cystogastrostomy opening combined with hydrogen peroxide irrigation can increase the success of endoscopic necrosectomy and decrease the number of required endoscopic interventions. The aim of the study is to assess the safety and feasibility of the technique in the management of WOPN.

**Methods:**

This is a retrospective chart review of all cases that underwent EUS with extended cystogastrostomy and hydrogen peroxide irrigation prior to necrosectomy in a tertiary referral medical center. Clinical success was defined as complete resolution of the cyst cavity or a cyst cavity less than 2 cm in size on follow-up imaging.

**Results:**

19 patients satisfied the inclusion criteria. The mean size of the walled-off cavity was 11 + 0.9 cm. Technical success of the procedure was 100%. The median number of necrosectomy sessions was 2 (range 1 to 7). Cavity resolution was noted in 18 out of 19 patients resulting in a clinical success of 94.7%. The median follow-up period was 12 months. The adverse events rate in our cohort was 15.7%.

**Conclusion:**

Extended cystogastrostomy coupled with hydrogen peroxide irrigation of WOPN cavity is safe and feasible.

## 1. Introduction

Walled-off pancreatic necrosis (WOPN) or infected pancreatic necrosis may develop in 40–70% of patients with pancreatic fluid collections following attacks of acute necrotizing pancreatitis [1]. Infected necrosis is frequently responsible for the late occurrence of multiple organ failure and sepsis and remains the major life-threatening complication of acute pancreatitis (mortality rate up to 30%). Median mortality rate of open surgical approaches can be up to 25% (range 12–56%) according to a recent systematic review [2]. High morbidity and mortality of laparotomy-based surgical intervention have necessitated the development of alternative therapies for the treatment of infected pancreatic necrosis.

Despite the overall success and application of endoscopic transmural techniques for pancreatic pseudocyst drainage, initial reports listed evidence of pancreatic necrosis as a contraindication for endoscopic intervention [3, 4]. Contrary to this belief, courageous investigators with surgical collaboration have evolved the endoscopic procedure to become widely applicable in patients with infected pancreatic necrosis. Baron et al. described the first reported experience of endoscopic treatment in pancreatic necrosis (*n* = 11) describing complications in up to 45% and recurrence at two years in 60% of the reported cohort after using stent drainage with nasocystic irrigation (mean procedure number = 2.7) [5].

Direct transmural endoscopic necrosectomy was first described by Seifert et al. in 2000 [6]. Nine years later, the same group published a large series from a multicenter trial (*n* = 93) which described an 84% success rate in the treatment of symptomatic pancreatic necrosis. This series described intervention over three procedures starting with initial transmural access and stent insertion and then balloon dilation (15–20 mm), followed by a third procedure for endoscopic removal of necrotic debris using forceful irrigation, suction, snares, forceps, and stone removal baskets. Repeated sessions were performed at intervals of 1–4 days until all necrotic material had been removed. Initial clinical success was 80% followed by sustained clinical improvement in 84% of included patients [7]. These serial treatments necessitate an extreme commitment of resources and expertise to treat each patient effectively.

Advances in endoscopic techniques and ultrasound guidance have made endoscopic cystogastrostomy and pancreatic necrosectomy the preferred options for treatment of infected pancreatic pseudocyst or walled-off pancreatic necrosis. Limitations of successful endoscopic drainage in these situations include the small size of the gastrostomy and the large amount of necrotic material within the cyst or abscess cavity. In this study we describe our technique of creating a large cystogastrostomy (2 cm) followed by hydrogen peroxide irrigation to facilitate drainage and necrosectomy. We propose that the use of extended gastrostomy and hydrogen peroxide may increase the success of direct endoscopic debridement and decrease the number of required endoscopic interventions for resolution of the abscess or necrosis cavity safely.

## 2. Methods

### 2.1. Study Design

This study represents a retrospective case series of patients referred to a single gastroenterologist and surgeon team at the University Medical Center of El Paso for management of acute pancreatitis complications. All patients with infected pseudocyst or walled-off pancreatic necrosis (WOPN) with or without infection who underwent Endoscopic Ultrasound (EUS) guided cyst necrosectomy with hydrogen peroxide irrigation were included (from August 2011 to April 2015). Patient with mature cavity wall and distance of less than 1 cm between the stomach wall and the cavity underwent this technique. Indications for endoscopic drainage in our cohort were infected pseudocyst or WOPN and symptomatic, noninfected WOPN which is larger than 6 cm and present with abdominal pain, gastric outlet obstruction, or jaundice. The clinical definition of acute pancreatitis complications was based on the revised Atlanta classification of acute pancreatitis [8]. The electronic database system (ProVation®, Minneapolis, MN) was used to identify the indication for the procedure, type of placed stent, number of sessions required for each patient, and adverse events. The electronic medical record was searched for patient demographics, imaging studies, and time for resolution of WOPN. The study was approved by the Institutional Review Board (IRB) of Texas Tech University Health Science Center.

### 2.2. Technique

**Figure 1:**
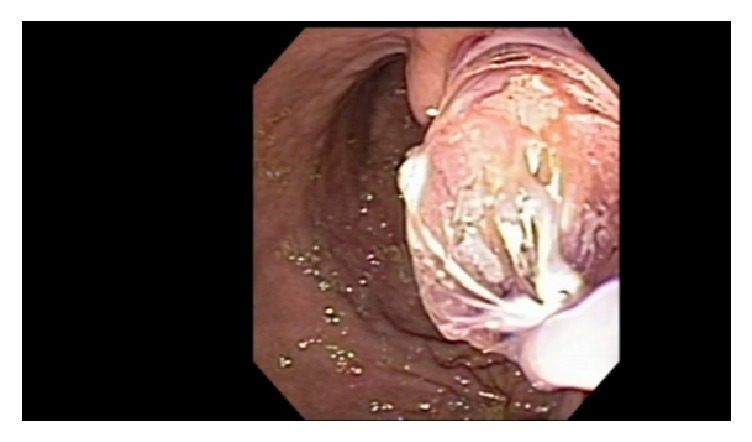
Balloon dilation of cystogastrostomy up to 20 mm.

**Figure 2:**
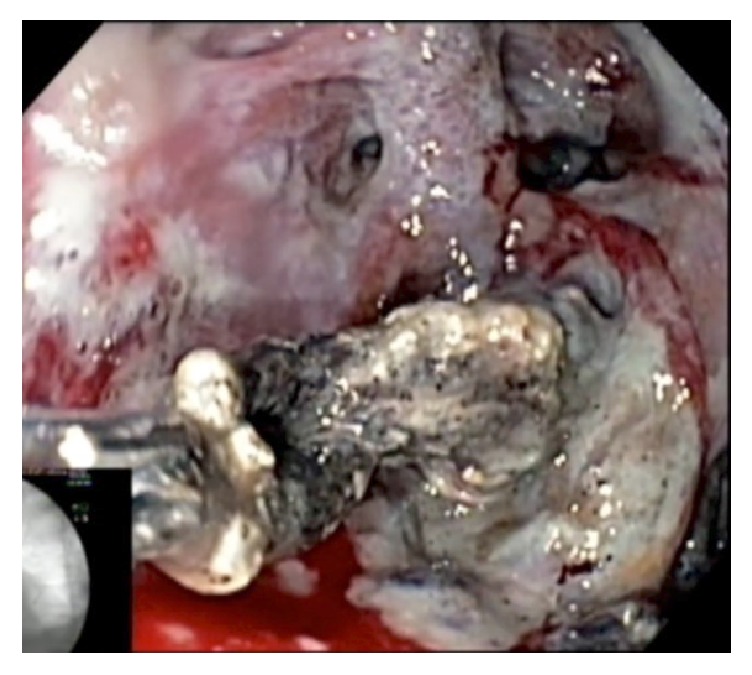
Removal of necrotic tissue after hydrogen peroxide irrigation.

**Figure 3:**
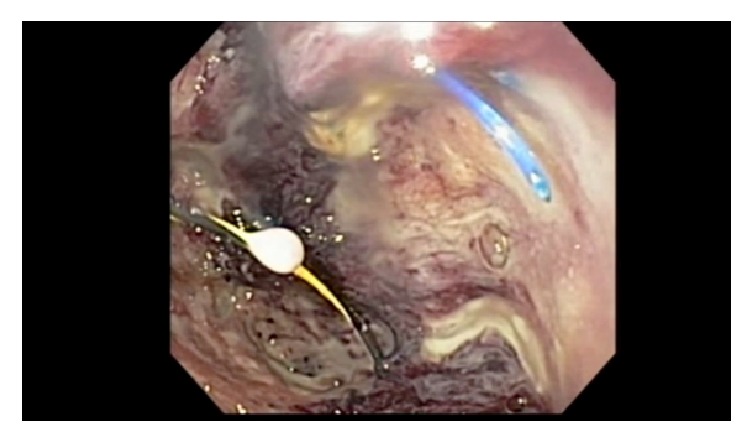
Surgical suture of distal pancreatectomy within pancreatic abscess bed.

**Figure 4:**
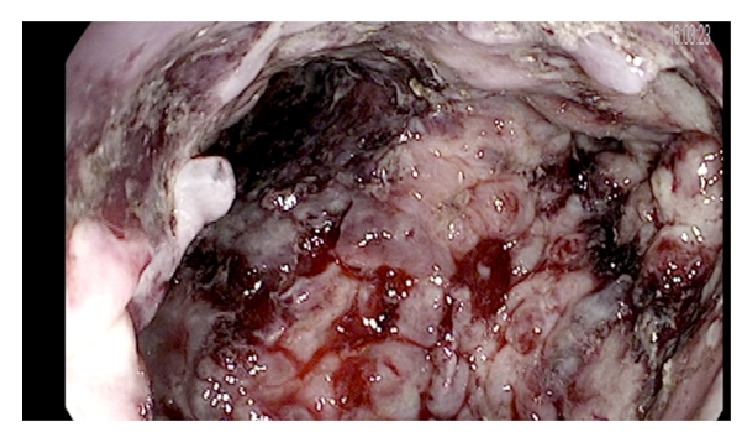
Walled-off pancreatic necrosis cavity after hydrogen peroxide irrigation and debridement.

This case series demonstrates a modified EUS-guided cystogastrostomy and necrosectomy technique. This technique involves the creation of an extended cystogastrostomy by single-stage dilation of the gastrostomy up to 2 centimeters if the EUS shows no blood vessels within the puncture site and the CT scan demonstrates a mature cyst wall. All procedures were performed by a single endoscopist (MO), who had performed more than 2000 EUS procedures at the time of the study. A linear Olympus GF-UC140P echoendoscope (Olympus America Inc., Center Valley, PA, USA) with a ProSound SSD 5000 processor (Aloka, Wallingford, CT, USA) was utilized in all cases. A 19-gauge flexible FNA needle Expect™ (Boston Scientific Natick, MA, USA) was advanced to the WOPN along the posterior wall of the stomach after ruling out intervening blood vessels using color Doppler. The stylet was then removed and cyst fluid was aspirated. A 0.035 in × 450 cm Jagwire (Boston Scientific, Marlborough, MA, USA) was then advanced through the needle and allowed to coil several times within the cyst under fluoroscopic guidance. The 19-gauge needle was then removed over the wire and needle knife (Boston Scientific, Marlborough, MA, USA) was introduced over the wire to create the cystogastrostomy using pure cut current. The gastrostomy was then dilated up to 20 mm using the scope CRE™ balloon dilator (Boston Scientific, Marlborough, MA, USA) (Figure 1). The echoendoscope was then removed over the guidewire and therapeutic gastroscope GIF-H180 (Olympus America Inc., Center Valley, Pa) was introduced alongside the wire and across the cystogastrostomy to the WOPN. Direct endoscopic examination of the cavity was done followed by hydrogen peroxide irrigation of the cavity using 30 cc of hydrogen peroxide mixed with 30 cc of water (1 to 1 concentration). Debridement of adherent necrotic tissue was done using several endoscopic retrieval devices such as nets, rat tooth forceps, and snares (Figures 2, 3, and 4). The final step included placement of two 10 French double pig tail stents or VIABIL® fully covered metal stent (CONMED, Utica, NY) based on the preference of the endoscopist to keep the gastrostomy tract open. The procedure was performed under general anesthesia administered by an anesthesiologist. Follow-up CT scan was done 2 to 4 weeks after the initial intervention. Endoscopic Retrograde Cholangiopancreatography (ERCP) was performed prior to cystogastrostomy if disconnected pancreatic duct was suspected on prior imaging. Stent removal was performed after confirmation of cavity resolution by an imaging study within 3 to 6 months from the initial debridement.

### 2.3. Statistical Analysis

Continuous data were reported with a mean and standard deviation or median with averages. Categorical data were reported as proportions. Technical success was defined as the ability to perform the previously described technique without failure in any of the described steps. Clinical success was defined as cyst cavity less than 2 cm in size on follow-up imaging.

## 3. Results

### 3.1. Patients Characteristics

A total of 19 cases (13 males and 6 females) satisfied the inclusion criteria with a mean age of 50 (±2.6) years. Etiology of the pancreatitis was gallstone in 12 patients, alcohol in 5 patients, and postsurgical (distal pancreatectomy) abscess and idiopathic in 1 patient each. The mean number of weeks from the index acute pancreatitis to the intervention was 9 (±2.2) weeks (range 2 to 52). Cases in this series were categorized as walled-off necrosis (WON) (7), infected WON (8), infected pseudocysts (3), and postpancreatectomy pancreatic abscess (1). Three patients had splenic vein thrombosis with extensive gastric varices on imaging. Three patients had multiorgan failure prior to the planned cystogastrostomy and necrosectomy (Table 1).

### 3.2. Procedure Characteristics

All cases were performed via a transgastric approach. The mean size of the walled-off cavity was 11 + 0.9 cm. Cystogastrostomy was created in 18 patients and it was dilated up to 20 mm. One patient had a wide open gastric fistula communicating with the WON and it was utilized for necrosectomy without the need for cystogastrostomy creation. ERCP was performed as a part of the evaluation for disconnected pancreatic duct in 9 patients (47.3%). Pancreatic duct stenting was done in 1 out of 9 patients for evidence of a pancreatic duct leak.

### 3.3. Study Outcomes

Technical success of the procedure was 100%. Median number of necrosectomy sessions was 2 (range 1 to 7). Cavity resolution was noted in 18 out of 19 patients resulting in a clinical success of 94.7%. The median follow-up period was 12 months (range 2 to 32 months). Radiological confirmation of cavity resolution in less than 3 months of the cystogastrostomy and necrosectomy was noted in 12 patients (63%). Among 18 patients who had cavity resolution, one patient required additional percutaneous drainage of paracolic gutter abscess. One patient died 5 days after the procedure from multiple organ failure (started prior to the procedure). As a result, the mortality rate of our series is 4.7%. Immediate adverse events were noted in one patient: bleeding from the gastrostomy site. Bleeding stopped after an epinephrine injection and fully covered stent placement. Delayed adverse events in the form of sepsis and recurrent cavity infection were seen in 2 patients. The two patients were treated with repeat cystogastrostomy and necrosectomy with hydrogen peroxide irrigation without any further adverse events. It was believed that cystogastrostomy blockage with a large amount of necrotic tissue was the culprit for recurrent infection in these patients. The complication rate in our cohort was 15.7% (3 out of 19 patients). One patient had a recurrence of fluid collection 18 months after cavity resolution. The fluid collection was 5 cm in size and it contained clear fluid. The fluid collection resolved with EUS and aspiration of the cavity fluid without further recurrence up to 30 months. No other cases of cyst recurrence were noted in our cohort (Table 2).

## 4. Discussion

In this manuscript, we illustrated our modified technique of creating extending cystogastrostomy followed by hydrogen peroxide irrigation for management of WOPN. The technique was successful in 100% of patients with complete cavity resolution noted in 95% of the cohort.

Necrotizing pancreatitis is complicated with serial episodes of sepsis and multiorgan failure. Attempts at open necrosectomy have demonstrated high morbidity and mortality necessitating the utilization of alternative techniques. The initial treatment or temporization of pancreatic necrosis with minimally invasive procedures such as percutaneous drainage (PCD), endoscopic necrosectomy (EN), and video assisted retroperitoneal debridement (VARD) has been reported as the Step-Up protocol in several multicenter trials to include the PANTER trial from the Dutch Group [9]. The PENGUIN trial demonstrated reduced complications in patients receiving endoscopic necrosectomy compared with open surgical debridement [10]. The American multicenter trial by Gardner et al. demonstrated similar results with reduced morbidity and mortality in the endoscopic debridement group [11]. The Dutch consensus group has developed the TENSION trial to directly compare outcomes from endoscopic drainage with alternative treatments to include PCD and VARD [12]. The preponderance of evidence demonstrates that endoscopic drainage has proven superior results to other therapeutic modalities in patient with walled-off pancreatic necrosis (WOPN). Recently, lumen opposing metal stent was proposed as a facilitator of endoscopic necrosectomy with great success in multiple series [13, 14]. In spite of the safety profile of the lumen opposing metal stents, life threating complications such as bleeding or perforation were reported in the literature [15].

The purpose of this study is to demonstrate that the creation of an extended cystogastrostomy at the initial endoscopic drainage followed by debridement assisted by hydrogen peroxide leads to reduced procedural interventions compared with techniques previously described in the literature. In our series, 63% of patients achieved complete resolution of WOPN within 3 months of the procedure in spite of the large size of the included WOPN. Hydrogen peroxide can work as a chemical debriding agent which aids in removing adherent necrotic tissue and irritates healthy tissue to induce the formation of granulation tissue and subsequent fibrosis with cavity resolution. The creation of a 2 cm cystogastrostomy opening facilitated the continuous drainage of the debrided necrotic tissue and accelerated the process of cavity resolution. Abdelhafez et al. reported similar outcomes for hydrogen peroxide irrigation in pancreatic debridement in a series of 10 cases. However, all cases in the above-mentioned series were performed without EUS guidance [16].

Oxygen embolism after hydrogen peroxide irrigation is reported in the literature [17]. However, most reported cases resulted from the hydrogen peroxide injection into closed cavities which leads to injection of hydrogen peroxide under high pressure forcing it to penetrate into capillaries and blood vessels [18]. We believe that we did not encounter any incident of oxygen embolism due to the creation of a large cystogastrostomy opening which prevented the injection of hydrogen peroxide under high pressure.

In our series, only one patient had cyst recurrence after complete endoscopic drainage. Disconnected pancreatic duct syndrome is a common cause for pancreatic fluid collection recurrence. Permeant transmural stenting was associated with a decrease in pancreatic fluid collection recurrence in this category of patients [19].

Challenges to the validity of this study include the retrospective nature of the study. There was no ability to achieve randomization since these patients were referred primarily for the presence of WOPN. This study was unable to determine the benefits of endoscopic debridement compared with surgical debridement since most patients had survived the first four weeks of necrotizing pancreatitis prior to arrival at the referral hospital. The majority of patients in our study had mature cyst wall with abutment to the stomach with a distance between the stomach and cyst wall of no more than one centimeter.

In conclusion, the creation of an extended cystogastrostomy followed by hydrogen peroxide irrigation was safe and feasible in endoscopic necrosectomy of WOPN and infected pseudocysts.

## Figures and Tables

**Table 1:** Patients characteristics.

Sex	Males: 13
	Females: 6

Age (mean ± SD)	50 (±2.6) years

Ethnicity	NHW: 11
	Hispanics: 8

Etiology of pancreatitis	Alcohol: 5
	Gallstone: 12
	Idiopathic: 1
	Postsurgical abscess: 1

Time from index AP to intervention (mean weeks ± SD)	9 (±2.2)

Type of fluid collected	WOPN: 7
	Infected WOPN: 8
	Infected pseudocyst: 3
	Pancreatic abscess: 1

Approach	Transgastric: 19

**Table 2:** Study outcomes.

Technical success (%)	100%
Clinical success (cavity resolution)	95.2 (18/19)
Median number of sessions (range)	2 (1–7)
Follow-up period (median, range)	12 (2–32)
Cavity resolution within 3 months	63%
Mortality rate	4.7% (1/19)
Adverse events rate	15.7 (3/19)
